# Editorial: The role of MicroRNAs and other non-coding RNAs in gut inflammation and gastrointestinal cancers

**DOI:** 10.3389/fmolb.2023.1173690

**Published:** 2023-03-20

**Authors:** Arivarasu N. Anbazhagan, Manmeet Rawat

**Affiliations:** ^1^ Division of Gastroenterology and Hepatology, Department of Medicine, University of Illinois at Chicago, Chicago, IL, United States; ^2^ Division of Gastroenterology and Hepatology, Department of Medicine, The Penn State University College of Medicine, Hershey, PaA, United States

**Keywords:** miRNA - microRNA, lnc RNA, gut inflammation, gastrointenstinal cancer, intestinal epithelia

The mammalian intestinal epithelium comprising of a single layer of cells is a highly dynamic structure with a diverse cellular composition (Allaire et al., 2018). The cells in the intestinal epithelium are organized in a crypt-villus structure with invaginations called ‘crypts of Lieberkühn’ where the intestinal stem cells responsible for regeneration reside. Rapidly regenerating intestinal epithelium signifies a perfect harmony among its various components essential for the maintenance of a perfect structure that serves many purposes-digestion, nutrient absorption, interaction with gut microbes and a harbor for immune cells (Morris and Choudhry, 2021). Recent evidence indicates that microRNAs (miR) play a crucial role in orchestrating these processes. Mice lacking *Dicer 1* (gene encoding microRNA processing enzyme) in intestinal epithelial cells exhibit marked changes in the epithelium with reduced number of goblet cells, increased crypt cell apoptosis and inflammation and impaired barrier (McKenna et al., 2010; Dhuppar and Murugaiyan, 2022). No doubt, several microRNAs regulating the expression of key intestinal genes have been reported. For example, miR-181 family works against colonic inflammation and epithelial cell injury (Jimenez et al., 2022), miR-31 is required for epithelial regeneration (Tian et al., 2017), miR-195, miR-802 and miR-429 are essential for goblet and Paneth cell function and several microRNAs are needed for adequate innate immune responses (Dhuppar and Murugaiyan, 2022). Moreover, microRNAs have emerged as key regulators of host and gut microbiota communication that is often disturbed in intestinal diseases (Dong et al., 2019). Thus, it is apparent that microRNAs are master regulators of gut homeostasis. Among other non-coding RNAs, the long non-coding RNAs (Lnc RNAs) with >200 nucleotides are also reported to significantly modulate cellular function in cell type-, tissue type- and differentiation stage-specific manner (Xiao et al., 2019). Several Lnc RNAs have been implicated in regulation of intestinal epithelial regeneration and barrier (Yarani et al., 2018; Xiao et al., 2019). The **Research Topic** brings forth some latest findings on this gut specific role of these non-coding RNA species.

Since their discovery in 1993 (Lee et al., 1993), microRNAs have been widely explored for their biomarker potential. They are not only expressed in tissues but also exist in biological fluids where their aberrant levels have been correlated with several diseases like gestational diabetes mellitus (Poirier et al., 2017) and colitis (Tili et al., 2017; Dhuppar and Murugaiyan, 2022). Fecal microRNAs secreted mainly by the intestinal epithelial cells (Liu et al., 2016) are a recent addition to the microRNA based diagnostic tools (Ahmed et al., 2009; Sarshar et al., 2020). Besides a biomarker for the disease condition fecal microRNAs can also indicate the gut microbiota health and regulation of dependent host functions (Viennois et al., 2019). These aspects along with the tools and techniques necessary for detection and quantification of fecal microRNAs have been covered in the review article by Rashid et al. The microRNAs in biological fluids and feces are released as component of the exosomes or from dying cells (Sarshar et al., 2020). These exosomal microRNAs by the virtue of availability to cells other than their source cells can modulate the function of the tissues in a paracrine and endocrine fashion. For example, miR-375 released into exosomes appearing from intestinal epithelial cells has been showb to target microbial tryptophanase gene in mice (Kumar et al., 2021). Enumerating a similar exosomal microRNA mediated distant tissue targeting in their elegant article Lian et al., show how intestinal exosomal miR-29b enriched in the circulation of ulcerative colitis (UC) patients is responsible for a compromised heart function observed in UC subjects. The importance of intestinal exosomal microRNAs in systemic and local tissue injury has also been highlighted in the excellent review by Park et al. Using sepsis as the disease model and aging as the aggravator of septic injury Park et al., hypothesize that basolaterally secreted gut exosomal microRNAs migrate to distant tissues to alleviate septic injury. It is fascinating to imagine how targeting gut exosomal machinery or microRNAs can help mitigate deregulation of peripheral tissue function that is induced by the disturbances in gut homeostasis.

The role of Lnc RNAs in intestinal disorders is gradually gaining prominence. Due to the added complexity of interaction of Lnc RNAs with microRNAs and RNA binding proteins and their tissue specific effects, the progress on deciphering role of Lnc RNAs in intestinal and other diseases is slow (Xiao et al., 2019). Non-etheless, their prognostic and diagnostic importance has led to steady efforts in the field (Yarani et al., 2018). In a similar quest, Liu et al., in their original research article have explored the TCGA database for differentially expressed Lnc RNAs in colorectal cancer patients identifying 6 Lnc RNAs of prognostic relevance. All articles covered in this Research Topic indicate the significance of studying non-coding RNAs. These RNA species not only can be therapeutic targets but also serves as efficient biomarkers of disease status. Intensive research on these applications, however, is needed to determine their clinical relevance. We hope you share our excitement in reading these articles enumerating some of these research efforts.

## References

[B1] Ahmed F. E. Jeffries C. D. Vos P. W. Flake G. Nuovo G. J. Sinar D. R. (2009). Diagnostic MicroRNA markers for screening sporadic human colon cancer and active ulcerative colitis in stool and tissue. Cancer Genomics - Proteomics 6, 281–295. 19996134

[B2] Allaire J. M. Crowley S. M. Law H. T. Chang S. Y. Ko H. J. Vallance B. A. (2018). The intestinal epithelium: Central coordinator of mucosal immunity. Trends Immunol. 39, 677–696. 10.1016/j.it.2018.04.002 29716793

[B3] Dhuppar S. Murugaiyan G. (2022). miRNA effects on gut homeostasis: Therapeutic implications for inflammatory bowel disease. Trends Immunol. 43, 917–931. 10.1016/j.it.2022.09.003 36220689 PMC9617792

[B4] Dong J. Tai J. W. Lu L. F. (2019). miRNA-microbiota interaction in gut homeostasis and colorectal cancer. Trends Cancer 5, 666–669. 10.1016/j.trecan.2019.08.003 31735285 PMC8480531

[B5] Jimenez M. T. Clark M. L. Wright J. M. Michieletto M. F. Liu S. Erickson I. (2022). The miR-181 family regulates colonic inflammation through its activity in the intestinal epithelium. J. Exp. Med. 219, 219. 10.1084/jem.20212278 PMC9462864 36074090

[B6] Kumar A. Ren Y. Sundaram K. Mu J. Sriwastva M. K. Dryden G. W. (2021). miR-375 prevents high-fat diet-induced insulin resistance and obesity by targeting the aryl hydrocarbon receptor and bacterial tryptophanase (tnaA) gene. Theranostics 11, 4061–4077. 10.7150/thno.52558 33754048 PMC7977461

[B7] Lee R. C. Feinbaum R. L. Ambros V. (1993). The *C. elegans* heterochronic gene lin-4 encodes small RNAs with antisense complementarity to lin-14. Cell 75, 843–854. 10.1016/0092-8674(93)90529-y 8252621

[B8] Liu S. da Cunha A. P. Rezende R. M. Cialic R. Wei Z. Bry L. (2016). The host shapes the gut microbiota via fecal MicroRNA. Cell Host Microbe 19, 32–43. 10.1016/j.chom.2015.12.005 26764595 PMC4847146

[B9] McKenna L. B. Schug J. Vourekas A. McKenna J. B. Bramswig N. C. Friedman J. R. (2010). MicroRNAs control intestinal epithelial differentiation, architecture, and barrier function. Gastroenterology 139, 1654–1664. 10.1053/j.gastro.2010.07.040 20659473 PMC3156097

[B10] Morris N. L. Choudhry M. A. (2021). Maintenance of gut barrier integrity after injury: Trust your gut microRNAs. J. Leukoc. Biol. 110, 979–986. 10.1002/JLB.3RU0120-090RR 33577717 PMC8357844

[B11] Poirier C. Desgagné V. Guérin R. Bouchard L. (2017). MicroRNAs in pregnancy and gestational diabetes mellitus: Emerging role in maternal metabolic regulation. Curr. Diab Rep. 17, 35. 10.1007/s11892-017-0856-5 28378294

[B12] Sarshar M. Scribano D. Ambrosi C. Palamara A. T. Masotti A. (2020). Fecal microRNAs as innovative biomarkers of intestinal diseases and effective players in host-microbiome interactions. Cancers (Basel) 12, 2174. 10.3390/cancers12082174 32764361 PMC7463924

[B13] Tian Y. Ma X. Lv C. Sheng X. Li X. Zhao R. (2017). Stress responsive miR-31 is a major modulator of mouse intestinal stem cells during regeneration and tumorigenesis. Elife 6, e29538. 10.7554/eLife.29538 28870287 PMC5584991

[B14] Tili E. Michaille J. J. Piurowski V. Rigot B. Croce C. M. (2017). MicroRNAs in intestinal barrier function, inflammatory bowel disease and related cancers-their effects and therapeutic potentials. Curr. Opin. Pharmacol. 37, 142–150. 10.1016/j.coph.2017.10.010 29154194 PMC5938753

[B15] Viennois E. Chassaing B. Tahsin A. Pujada A. Wang L. Gewirtz A. T. (2019). Host-derived fecal microRNAs can indicate gut microbiota healthiness and ability to induce inflammation. Theranostics 9, 4542–4557. 10.7150/thno.35282 31285778 PMC6599659

[B16] Xiao L. Gorospe M. Wang J. Y. (2019). Long noncoding RNAs in intestinal epithelium homeostasis. Am. J. Physiol. Cell Physiol. 317, C93-c100. 10.1152/ajpcell.00092.2019 31042423 PMC6689755

[B17] Yarani R. Mirza A. H. Kaur S. Pociot F. (2018). The emerging role of lncRNAs in inflammatory bowel disease. Exp. Mol. Med. 50, 1–14. 10.1038/s12276-018-0188-9 PMC6283835 30523244

